# Biochemical and Molecular Profiling of Wild Edible Mushrooms from Huila, Angola

**DOI:** 10.3390/foods11203240

**Published:** 2022-10-17

**Authors:** Raquel Kissanga, Ângela Liberal, Inês Diniz, Ana S. B. Rodrigues, João L. Baptista-Ferreira, Dora Batista, Marija Ivanov, Marina Soković, Isabel C. F. R. Ferreira, Ângela Fernandes, Lillian Barros, Luís Catarino

**Affiliations:** 1 Centre for Ecology, Evolution and Environmental Changes (cE3c), CHANGE—Global Change and Sustainability Institute, Faculty of Sciences, University of Lisbon, Campo Grande, 1749-016 Lisbon, Portugal; 2 Departamento de Biologia, Faculdade de Ciências, Universidade Agostinho Neto, Av. 4 de Fevereiro 71, Luanda 999104, Angola; 3 Centro de Investigação de Montanha (CIMO), Instituto Politécnico de Bragança, Campus de Santa Apolónia, 5300-253 Bragança, Portugal; 4 Laboratório Associado para a Sustentabilidade e Tecnologia em Regiões de Montanha (SusTEC), Instituto Politécnico de Bragança, Campus de Santa Apolónia, 5300-253 Bragança, Portugal; 5 Linking Landscape, Environment, Agriculture and Food (LEAF), TERRA—Associated Laboratory for the Sustainability of Land Use and Ecosystem Services, Instituto Superior de Agronomia, University of Lisbon, Tapada da Ajuda, 1349-017 Lisboa, Portugal; 6 BioISI—Biosystems & Integrative Sciences Institute, Faculty of Sciences, University of Lisbon, Campo Grande, 1749-016 Lisbon, Portugal; 7 Institute for Biological Research “Siniša Stanković”—National Institute of Republic of Serbia, Department of Plant Physiology, University of Belgrade, Bulevar Despota Stefana 142, 11000 Belgrade, Serbia

**Keywords:** fungi, wild edible mushrooms, nutritional value, chemical composition, molecular identification

## Abstract

The harvesting, processing, and sale of wild edible mushrooms (WEM) is a relevant economic activity in Angola and a good example of the use of non-wood forest products for food. Although there is deep traditional knowledge about the general properties of WEMs, a huge gap remains in detailed scientific knowledge. Thus, this study aimed to investigate the socio-economic importance of the species sold at local markets in Huila, Angola, from their molecular identification to the assessment of their nutritional, chemical, and bioactive profiles. From the eight WEM morphotypes studied, five were identified based on phenotypical and molecular approaches (four *Russula* spp., and *Amanita loosei*). The studied mushrooms proved to be a rich source of carbohydrates, proteins, and ashes, also presenting low amounts of fat. Chemical analyses further revealed mannitol as the main free sugar in all samples, and organic acids, namely, oxalic, quinic, malic, citric, and fumaric acids in low amounts. Additionally, the α-tocopherol isoform and monounsaturated fatty acids were predominant. Regarding phenolic acids, protocatechuic, *p*-hydroxybenzoic, *p*-coumaric, and cinnamic acids were detected in all mushroom hydroethanolic extracts, being responsible for their antioxidant, antibacterial, and antifungal activities. Our investigation contributes to the identification and knowledge of WEMs as important complementary food sources in Angola, some of which were reported for the first time, promoting their utilization as a basis of nutritional and functional ingredients, as being able to be part of a balanced diet and to be used in new bio-based formulations.

## 1. Introduction

For centuries, wild edible mushrooms (WEMs) have been collected from forests, or later cultivated and consumed, due to their nutritional value, medicinal utility, and unique flavour [1]. In recent decades, worldwide research on the use of WEMs has gained ground as their consumption and commercialization in local businesses proved to be important for the rural subsistence of developing countries [2,3,4]. However, some cultures avoid their consumption due to religious beliefs, fear of poisoning, and the fact that habitats where wild mushrooms grow may contain decomposing matter [5].

Fresh and preserved mushrooms are consumed in many countries as a delicacy, particularly for their unique aroma, flavour, and texture [2]. Worldwide, WEMs are known to be a healthy source of food with valuable nutritional qualities, such as their richness in carbohydrates, proteins, fibers, vitamins (B and C complexes) and minerals, and the presence of unsaturated fatty acids, mainly linoleic acid, and low-fat totals, which makes them an excellent food product to be included in low-calorie diets [6,7]. The proteins that are commonly part of mushrooms hold all the essential amino acids for human nutrition, being mainly rich in lysine and leucine, normally absent from cereal-based foods [8,9], present in an easily digested form [8,9,10]. In addition, mushrooms are seen as functional foods and/or bases of nutraceuticals, given the presence of physiologically and biologically active substances such as phenolic acids [11], largely responsible for their strong antibacterial and antifungal activities, often with superior antimicrobial responses to frequently used drugs [12,13]. Knowledge about the uses and properties of WEMs has generally been passed from one generation to another by word of mouth and without written documentation. Although ethnomycological knowledge among African communities has been addressed in several published works [14,15,16], which document a wide range of traditional uses of WEMs for food and medicinal purposes, this subject remains largely unstudied. In many Tropical African countries, WEMs are an important food source, being largely consumed fresh in the periods of fruiting bodies development or dried along the year. Several works have been published on the chemical composition and nutritional value of WEMs in African countries demonstrating its valuable nutritional, medicinal, functional, and nutraceutical properties [17,18,19,20,21]. Thus, the deepening of knowledge about the taxonomy, ecology, traditional uses, food, and functional properties of WEMs contributes to the food security of rural communities and a way to promote the sustainable use of natural resources [3].

Correctly identifying mushrooms is challenging [22] as no single tool can allow unambiguous species identification in most cases [23]. In recent decades, mycologists have classified mushroom species based on morphology, niche types, growth behavior, interaction with the host, and ecological factors, which, in many cases, are insufficient for accurate identification [24]. An integrative taxonomical approach seems to be the best practice for species identification, aiming at evaluating diagnostic characters, either phenotypic, molecular, or both, also combining genealogy (phylogeny), phenotype (including autecology), and reproductive biology data (when feasible). In the last decade, molecular biology techniques have been increasingly used to identify unknown samples and cryptic species based on current classification. Although there is currently no single tool for identifying fungi, DNA barcoding has succeeded in identifying between and within several fungi genera [22]. The ITS region is the most widely analyzed for fungal species identification, namely macro-fungi, remaining the first diagnosis at the genus level [23]. However, when this ribosomal RNA region is coupled with other marker genes, multi-locus fungal identification is achieved with an enhancement of identification resolution. Several gene makers have been analyzed in molecular studies of Russula, with multiple genes being identified as good intra- and inter-specific markers. Secondary DNA barcodes, such as RPB1, RPB2, and LSU, are being increasingly implemented for taxonomic groups where ITS does not provide sufficient distinctiveness [23].

In Central and Southern Africa, the collection of WEMs for consumption is an important activity that contributes both to the food security of local populations and to the livelihoods of many families [24,25,26,27]. Edible mushrooms are exploited for food and trade by local populations also in the miombo area [28,29], but very little information is available for Angola. For instance, in “The Edible Fungi of Tropical Africa” annotated database [27], only 24 harvests are registered for Angola, in the provinces of Kwanza Sul and Benguela, belonging to 11 species: *Cantharellus congolensis* Beeli [30], *C. defibulatus* (Heinem.) Eyssart and Buyck, *C. densifolius* Heinem., *C. ruber* Heinem., *C. splendens* Buyck [31], *C. sublaevis* Buyck and Eyssart., *Gyrodon miretipes* Heinem. and Rammeloo, *Mackintoshia persica* Pacioni and C. Sharp [32], *Russula congoana* Pat., [32], *R. flavobrunnea* Buyck [33], and *R. phaeocephala* Buyck [33]. Many species of non-timber forest products (NTFP) and their derivatives are currently used in this country [34,35,36,37,38], with many WEMs being collected and sold at roadsides, local markets, and city markets, including in the capital, Luanda. However, despite its large socio-economic importance, there is very little information on the fungi species occurring or being used in Angola [27,39,40], nor about the identification and properties of WEMs collected and traded. In the province of Huila, southwest Angola, rural communities collect a wide variety of mushrooms, both for self-consumption and for informal sales, particularly in Lubango, the capital of the province, and neighboring municipalities. In the city markets, dried mushrooms are sold throughout the year, whereas fresh mushrooms are sold only at harvest time.

Thus, the main objectives of this study were: (i) to characterize the socio-economic relevance of the mushroom trade and consumption in Huíla (Angola); (ii) to identify the mushroom species in the dried mixtures sold in the Humpata market, Huila, based in morphological and molecular data; and (iii) to document their chemical, nutritional and functional properties.

## 2. Materials and Methods

### 2.1. Markets and Mushroom Material

**Figure 1:**
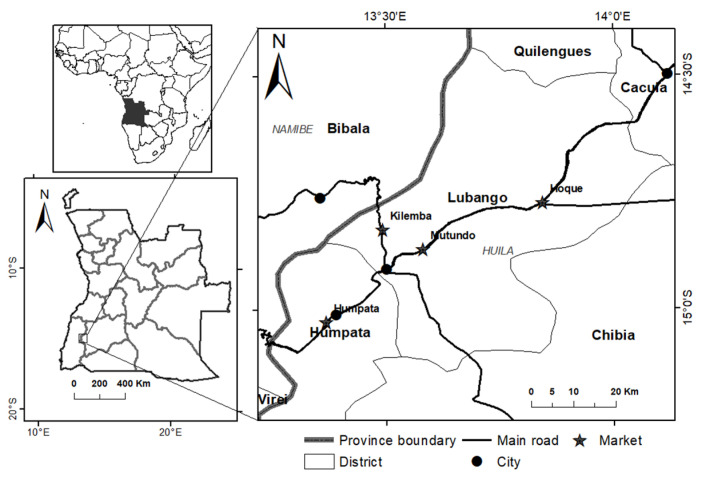
Location of the study site in Angola.

The field prospection took place between June and October 2018 at the markets of Lubango and surrounding municipalities: Mutundo, Humpata, Hoque, and Rio Nangombe (Figure 1). The selling prices of the dried mushroom mixtures were documented, and 5 replicates of each selling unit were weighed. In addition, sales prices and weights of fresh mushrooms were recorded at each market at the beginning of the day. Samples of the dried mushroom mixtures were purchased from the Humpata municipal market, the only place where sufficiently intact samples could be acquired for the accurate separation of morphotypes. With the cooperation of selected sellers, the mushroom morphotypes present in the samples were identified, separated, and numbered, and their common names were recorded in the two main local languages used in Huila, Umbundu, and Nyaneka. Information regarding harvest sites, the ecology of the species, mushroom picking and drying processes was also documented through visits to the collecting sites.

### 2.2. Morphological Characterization

Mushroom samples were stored in dry conditions, and macromorphological features and field characters were recorded at the collecting location. According to these features, eight different morphotypes were separated and numbered from M1 to M8. As measurements on fresh material were not taken in the field, cap and stipe dimensions were taken on the dried specimens for comparison. Micromorphological characters were observed from small tissue pieces of lamellae of the dried material mounted in a mixture of 5% KOH and 1% Congo red with the help of a compound microscope (Olympus BX50). Staining of basidiospores was performed by chemical reaction with Melzer’s reagent. The following macro- and micromorphological parameters were recorded: Cap: color, surface aspect, and diameter in cm; Stipe: color, diameter and length in cm; Spores: shape, surface morphology, and dimensions in μm; Basidia: dimensions in μm.

Available bibliography on mushrooms in southern and tropical Africa as well as websites with relevant information on the mushroom species that occur in the region were used to obtain information on the African mushrooms, particularly on edible species [25,26,27,28,29].

### 2.3. Molecular Analysis

#### 2.3.1. DNA Extraction, Amplification, and Sequencing

Individual specimens of samples M1 to M8 were ground into power with liquid nitrogen and stored at −80 °C until further analysis. Total genomic DNA was extracted with the commercial kit DNeasy plant mini kit (Qiagen, Germany) according to the manufacturer’s instructions. DNA concentration and purity were measured at 260/280 nm and 260/230 nm in a Multiskan SkyHigh Microplate Spectrophotometer (Thermofisher scientific, Waltham, MA, USA). Amplification of DNA was performed in a MyCycler Thermal Cycler (Bio-rad, Hercules, CA, USA), in a polymerase chain reaction (PCR) mixture containing 2 µL genomic DNA (10 ng/µL), 4 µL of 5 × GoTaq G2 Flexi buffer (Promega, Madison, WI, USA), 0.5 µL of dNTP (Fermentas, Waltham, MA, USA) (0.2 mM), 2 µL of MgCl_2_ (1.5 mM) (Promega, Madison, WI, USA), 1 µL of each primer (1 mM) (Stabvida, Caparica, Portugal), 0.2 µL of GoTaq G2 Flexi DNA (5 U/µL) (Promega, Madison, WI, USA), and sterile ultrapure water. Three nuclear markers were amplified as described: (1) the internal transcribed spacer region of ribosomal DNA (ITS), comprising the ITS1 and ITS2 spacer regions and the ribosomal gene 5.8 S, using primers ITS1 and ITS4 [41]; (2) a part of the ribosomal large subunit 28 S region (LSU), using primers LR0R and LR5 [42]; (3) the region between the conserved domains 6 and 7 of the second largest subunit of the RNA polymerase II (rpb2), using primers bRPB2-6F and bRPB2–7.1R [43]. PCR products were visualized by electrophoresis in 1,2% agarose gel in an omniDOC Gel (Cleaver scientific, Rugby, UK). PCR products were purified with SureClean (Bioline, Alvinston, ON, Canada) according to the manufacturer’s instructions and sequenced by Stabvida (Caparica, Portugal). Sequences were edited with DNASTAR lasergene version 11.1.0.54 (Madison, WI, USA) and deposited in GenBank (accession numbers in Table S1).

#### 2.3.2. Phylogenetic Analyses

Generated sequences from all samples (M1-M8) were queried against nucleotide sequences available in the NCBI database, using BLASTn 2.10.0+ [44], directly on the NCBI webpage (https://blast.ncbi.nlm.nih.gov (accessed on 11 July 2022)). Those with the most significant hits, as well as species representative sequences from previous phylogenetic works and from type-specimens, whenever available, were retrieved from GenBank (78, 92, and 91 sequences of ITS, LSU, and rpb2, respectively) and included in the phylogenetic analyses. A total of 99 individuals (five specimens of Amanita spp and 94 specimens of Russula spp) were analyzed. Reference publications and accession numbers for the sequences employed are provided in the Supplementary Table S1.

Sequences for each DNA region were aligned with MAFFT version 7.453 [45] and then examined manually using the AliView version 1.25 [46]. The alignment matrices were converted to the appropriate format and concatenated using the TriFusion V0.4.12 software (https://github.com/OdiogoSilva/TriFusion (accessed on 24 May 2022)). Phylogenetic relationships using Maximum Likelihood (ML) and the Bayesian inference (BI) methods were inferred using a concatenated alignment matrix (ITS, LSU, and rpb2). For ML analysis, RAxML version 8.2.12 [47] was used with unlinked partitions corresponding to each DNA region and the GTRCAT nucleotide substitution model. Branch support was estimated by performing 1000 bootstrap replicates. The BI analyses were conducted in MrBayes version 3.2.6 [48] and two runs were performed applying the Monte Carlo Markov Chain (MCMC) method iterated for 4,000,000 generations, with a sampling frequency of 4000 generations. A 50% majority rule consensus tree was obtained after discarding the first 25% of trees. TRACER version 1.7.1 [49] was used to verify the files and ensure that the chains had reached convergence. A GTR + GAMMA model of sequence evolution was applied to each DNA partition, as estimated by the software jModelTest2 [50] using the corrected Akaike Information Criterion (AICc) [51].

### 2.4. Extract Preparation

The samples of ground powdered mushrooms (2 g) were twice stirred with ethanol: water (80:20 *v*/*v*; 30 mL) at 25 °C and 150 rpm for 1 h, with subsequent filtration through Whatman paper no. 4. The two combined fractions were then evaporated using a rotary evaporator (Büchi R-210; Flawil, Switzerland) at 40 °C under reduced pressure, to evaporate the ethanolic portion of the extract. The obtained aqueous extracts were finally frozen at 4 °C and freeze-dried for further analysis [52].

### 2.5. Chemical Characterization

#### 2.5.1. Nutritional and Energetic Value

The nutritional composition of the mushroom samples was performed using AOAC procedures [53], relating to its configuration in proteins, fat, carbohydrates, and ashes. Crude protein content (*N* × 4.38) was estimated using the macro-Kjeldahl method; the crude fat quantity was determined by extracting a known weight of sample with petroleum ether using a Soxhlet apparatus; the ash content was determined by incineration at 550 ± 15 °C. The total carbohydrate quantity (g per 100 g of dried weight (dw)) was calculated by difference and total energy given to the next equation: Energy (kcal/100 g dw) = 4 × (g protein + g carbohydrates) + 9 × (g fat).

#### 2.5.2. Hydrophilic Compounds

Free individual sugars were measured by high-performance liquid chromatography coupled to a refraction index detector (HPLC-RI, Knauer, Smartline system 1000; Berlin, Germany), as previously described by Spréa et al. [54]. Briefly, dried sample powder (1 g) was spiked with melezitose as internal standard (IS, 5 mg/mL), and was extracted with 40 mL of 80% aqueous ethanol at 80 °C for 30 min. The resulting suspension was centrifuged at 30× *g* for 10 min. The supernatant was concentrated at 60 °C under reduced pressure and defatted three times with 10 mL ethyl ether, successively. After concentration at 40 °C, the solid residues were dissolved in water to a final volume of 5 mL and filtered through 0.2 μm nylon filters from Whatman. Identification was achieved by comparing the relative retention times of sample peaks with standards under the same chromatographic conditions, and the obtained data were analyzed using Clarity 2.4 Software (DataApex, Podohradska, Czech Republic). The internal standard (IS, raffinose) method was used, and the quantification was based on the RI signal response of each standard. The results were expressed in g per 100 g of dw.

Organic acids were assessed using a Shimadzu 20 A Series UFLC (Shimadzu Corporation, Kyoto, Japan), as previously described [55]. Samples (~2 g) were extracted by stirring with 25 mL metaphosphoric acid (25 ° C at 30× *g*) for 45 min and subsequently filtered through Whatman No. 4 paper. Before analysis, the sample was filtered through 0.2 μm nylon filters. The detection was performed using a diode array detector (DAD), at 215 nm as preferred wavelengths. The organic acids found were quantified using calibration curves obtained for each commercial compound. The results were expressed in g per 100 g of dw.

#### 2.5.3. Lipophilic Compounds

The tocopherol content was evaluated as previously described [54]. BHT solution in hexane (10 mg/mL; 100 μL) and internal standard (IS) solution in hexane (tocol; 50 μg/mL; 400 μL) were added to the sample prior to the extraction procedure. The samples (∼500 mg) were homogenized with methanol (4 mL) by vortex mixing (1 min). Subsequently, hexane (4 mL) was added and again vortex mixed for 1 min. After that, saturated NaCl aqueous solution (2 mL) was added, the mixture was homogenized (1 min), centrifuged (5 min, 4000× *g*) and the clear upper layer was carefully transferred to a vial. The sample was re-extracted twice with hexane. The combined extracts were taken to dryness under a nitrogen stream, redissolved in 2 mL *n*-hexane, dehydrated with anhydrous sodium sulfate, filtered through 0.2 μm nylon filters from Whatman, transferred into a dark injection vial and analysed using an HPLC system (Knauer, Smartline system 1000; Berlin, Germany) coupled with a fluorescence detector (FP-2020; Jasco, Oklahoma City, OK, USA). Individual compounds were identified by comparison with authentic standards under the same chromatographic conditions. By using the internal standard method (IS), the quantification was based on the fluorescence signal response, and the tocopherol content was expressed in μg per 100 g of dw.

Fatty acid methyl esters (FAME) were investigated after trans-esterification of the lipid fraction attained through Soxhlet extraction as previously described [54]. Fatty acids were methylated with 5 mL methanol–sulfuric acid–toluene 2:1:1 (*v/v/v*), during at least 12 h in a bath at 50 °C and 30× *g*; then 3 mL deionized water was added to obtain phase separation; the FAME were recovered with 3 mL diethyl ether by shakingin a vortex, and the upper phase was passed through a micro-column of anhydrous sodium sulfate in order to eliminate the water; the sample was recovered in a vial with Teflon, and before injection the sample was filtered with a 0.2 μm nylon filter from Whatman. The analysis was performed by gas–liquid chromatography with flame ionization detection, using a YOUNG IN Chromass 6500 GC System instrument equipped with a *split/splitless* injector, a flame ionization detector (FID) and a Zebron-Fame column. The identification and quantification of fatty acids were achieved by comparing the relative retention times of FAME peaks from samples with standards (standard mixture 47885-U, Sigma, St. Louis, MO, USA) and results were recorded and processed using the Software Clarity DataApex 4.0 Software (Prague, Czech Republic) and expressed in relative percentage of each fatty acid.

### 2.6. Determination of Phenolic Acids and Related Compounds, and Bioactive Properties

#### 2.6.1. Phenolic Acids and Related Compounds

Phenolic acids and related compounds were analyzed with UFLC equipment coupled to a diode array detector (DAD) [56], after dissolving the hydroethanolic extracts in 20% aqueous methanol, at a known concentration. The phenolic acids and related compounds were quantified by comparison of the area of their peaks recorded at 280 nm, with calibration curves obtained from commercial standards. The results were expressed in µg per g of extracts.

#### 2.6.2. Antioxidant Activity

The antioxidant activity was evaluated by a cell-based procedure, namely, through the inhibition of the production of thiobarbituric acid reactive substances (TBARS). For this assay, the lyophilized hydroethanolic extracts were dissolved in water and subjected to dilutions from 5 to 0.625 mg/mL. Lipid peroxidation inhibition in porcine (*Sus scrofa*) brain homogenates was estimated by the diminution in TBARS; the color strength of malondialdehyde–thiobarbituric acid (MDA–TBA) was measured by its absorbance at 532 nm; the inhibition ratio (%) was calculated using the following formula: [(A − B)/A] × 100%, where A and B were the absorbances of the control and the sample solutions, respectively [57]. The results were expressed in EC_50_ values (mg/mL, sample concentration providing 50% of antioxidant activity). Trolox was used as a positive control.

#### 2.6.3. Antimicrobial Activity

The extracts were redissolved in 5% dimethyl sulfoxide (DMSO) to a concentration of 10 mg/mL and further diluted. The microdilution method [58] was performed to assess the antimicrobial activity against the Gram-negative bacteria *Escherichia coli* (ATCC 25922), *Salmonella typhimurium* (ATCC 13311) and *Enterobacter cloacae* (ATCC 35030), and Gram-positive bacteria: *Staphylococcus aureus* (ATCC 11632), *Bacillus cereus* (clinical isolate), *Listeria monocytogenes* (NCTC 7973). For antifungal assays, six micromycetes were used: *Aspergillus fumigatus* (human isolate), *Aspergillus niger* (ATCC 6275), *Aspergillus versicolor* (ATCC11730), *Penicillium funiculosum* (ATCC 36839), *Trichoderma viride* (IAM 5061) and *Penicillium verrucosum* var. *cyclopium* (food isolate). The minimum extract concentrations that completely inhibited bacterial growth (MICs) were determined by a colorimetric microbial viability assay, and minimum bactericidal concentration (MBC) and minimum fungicidal concentration (MFC) were also calculated. Streptomycin, ampicillin, ketoconazole, and bifonazole (Sigma-Aldrich, St. Louis, MO, USA) were used as positive controls, and 5% DMSO was used as a negative control.

### 2.7. Data Analysis

Eight mushroom samples were analyzed for all the experiments, and the antioxidant assays were carried out in triplicate. The results are expressed as mean values ± standard deviation (SD). The differences between samples were analyzed using one-way analysis of variance (ANOVA) followed by Tukey’s honestly significant difference post hoc test with α = 0.05, coupled with Welch’s t-test. This analysis was carried out using the SPSS v. 22.0 program.

A Principal Component Analysis (PCA) was performed to evaluate if chemical and functional characteristics could separate the samples analyzed. As they were measured in different units, PCA was applied to standardized variables (centered by the mean and scaled by the variance). The analysis was performed in R version 3.6.3 (R Core Team, 2017) using the “prcomp” function from the “stats” package, and figures were produced using the package “ggplot2” version 3.2.1 [59].

## 3. Results and Discussion

### 3.1. Prices, Quantities Sold, and Socio-Economic Importance

The harvesting, processing, and sale of WEMs is an important economic activity in Huíla, and the consumption of edible mushrooms is a good example of the use of non-wood forest products in human nutrition. During prospection, 35 mushroom vendors were found in the four markets visited: 15 at Mutundo market, 10 at Humpata, 6 at Hoque, and four at Rio Nangombe. In total, 21 vendors were interviewed: 10 at Mutundo market, 5 at Humpata, and 3 at Hoque and Rio Nangombe markets each. The selling units and prices for dried mushrooms do not vary much among vendors, who tend to sell the products in the same containers or arrange them in similarly shaped piles. Vendors adopt a characteristic medium-sized dish that may contain 65–120 g of dried mushrooms, with an average of ~90 g. Additionally, the prices practiced are uniform in each market. In all markets, the price per unit is AKZ 100, except in the Hoque market, where the unit was sold at AKZ 150 (0.20 and 0.30 USD/kg, respectively, considering the official exchange rate of the Bank of Angola, at the date of the fieldwork, USD 1 = AKZ 500). As for fresh mushrooms, they are sold in bowls or large plates, and prices vary among markets and also throughout the day in the same market. As they are perishable products, it is common for vendors who still have fresh mushrooms in the evening to reduce the price to sell them all. The prices recorded in the morning varied between AKZ 100 and 500 for quantities between 200 and 1500 g. When converted to price per kilogram, the dried mushrooms were sold between AKZ 1322 and 1741 (2.64 and 3.48 USD/kg, respectively) and the fresh mushrooms between AKZ 250 and 333 (0.50 and 0.67 USD/kg, respectively). The recorded selling prices for dried WEMs make them an affordable food source that can contribute to food security in both rural and urban communities, as well as a source of income for households. In the Huila markets, the prices of dried mushrooms seem to have little variation along the year and among markets. Conversely, the prices of fresh mushrooms may vary during the day because they cannot be stored until the next day, and presumably also throughout the year, as the abundance of mushrooms varies and is related to the climatic conditions, namely, the occurrence of rains.

Regarding the quantity of mushrooms sold daily per seller, there is great variability but, with the information that was possible to obtain, each vendor may sell about 900 g of dried mushrooms per day. Knowing that the dried mushrooms are sold throughout the year and considering the 35 vendors that resulted from the census, we can consider that in the four markets will be sold about 31.5 kg of dried mushrooms per day, which corresponds to 11.3 tons per year. Each vendor interviewed can earn at least AKZ 900 daily (c. USD 1.8) from the sale of mushrooms and thus contribute to the family income of around AKZ 21,600 per month (USD~43.2), considering 24 days of sales per month. On the other hand, it is not possible, with the data obtained, to make an accounting regarding fresh mushrooms. These are sold according to the maturation of the fruiting bodies, which depends on the occurrence of rainfall. Additionally, the sale of fresh mushrooms is largely carried out along the roads by informal vendors, often children, and prices vary greatly both throughout the year and during the day.

The mushrooms sampled in the present study are mycorrhizal and abundant in the Miombo forests in the central highlands of Huila, in association with key tree species, particularly Julbernardia, Brachystegia and Uapaca, occurring alone or in groups. They can be found both on litter-covered soils from the shadiest sites under the tree canopy, as well as in more open sites with rocky soil and herbaceous or shrubby strata on sloping terrain. Although spore germination occurs in the rainy season, the production of fruiting bodies is highly dependent on the intensity and frequency of rainfall throughout the year.

**Figure 2:**
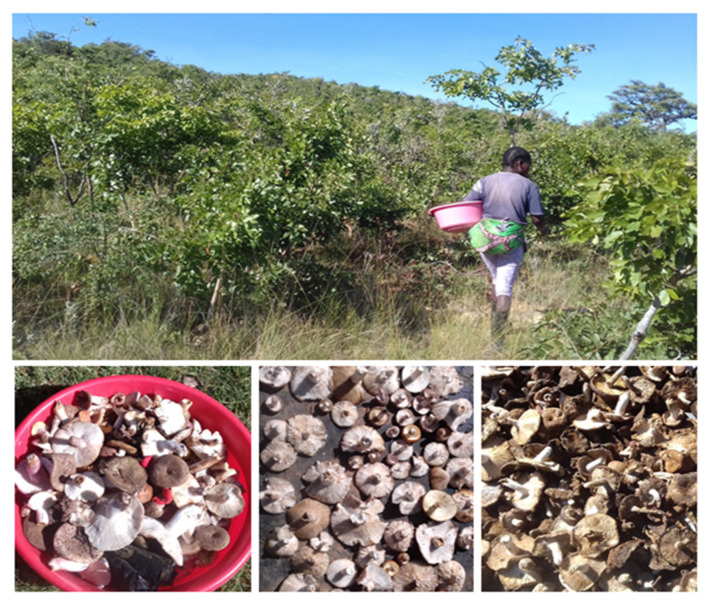
Mushrooms picking in a Miombo woodland area (**top**) and mixtures of fresh edible mushrooms, and mushrooms at different stages of drying (**bottom**). Photos by Herose Mendes.

Eight morphotypes of mushrooms were recognized in the mixtures sold at the Humpata market and separated and numbered as M1 to M8. Through the visit to the harvesting sites, it was observed that the WEMs are harvested in forest vegetation of Miombo and Mopane, as well as fallow land and forest areas cleared for cultivation, with soil being the predominantly substrate. The mushrooms, usually of a mixture of species, are air-dried until they are brittle, the moisture reduced (Figure 2), and then packaged and stored.

The mushroom species identified correspond to the ones present in the mixtures sold at the Humpata market at the time of acquisition. Several other species are sold both fresh and dried in the city markets and along roadsides. In fact, during the fieldwork, it was possible to observe several other species of mushrooms that are harvested and sold fresh, namely, of the genera *Amannita*, *Cantharellus*, *Lactarius* and *Termitomyces.* However, according to information obtained from local informants, such species are not suitable for drying and long-term preservation, probably because they have high moisture contents and degrade before drying.

Despite the socio-economic importance and scientific interest, the knowledge about the diversity and properties of macrofungi in Angola is very incipient, with only 24 occurrences of edible mushrooms recorded, belonging to 11 species of four genera [27]. None of them coincides with the species identified in this work, although the genus *Russula* is well represented in both cases. Additionally, no accessions from Angola were found in DNA sequence databases, and no publications are available on the subject. The information available for neighboring countries to Angola, where many dozens of edible WEM species are known [25,26,27], allows us to infer that there is a great diversity of edible fungi in Angola too, but it is still almost totally unknown. In fact, it is very frequent to find a great variety of fresh and dried mushrooms for sale all over Angola, both in the markets and at the roadside.

### 3.2. Morphological Characterization and Molecular Identification of Sampled WEM

With the purpose of identifying the collected mushroom morphotypes up to the species level and improving the knowledge on these local food resources, a comprehensive morphological and molecular characterization was performed. Table 1 summarizes the set of observations made on the mushroom samples, supplemented with data from the vernacular names and ecological data obtained during the fieldwork campaign, and the molecular characterization performed in this study. By combining field information and the observations made at the laboratory on the dry material, samples M1 to M7 were recognized as belonging to the Russulaceae family and *Russula* genus. The presence of ring and volva revealed sample M8 to be an Amanitaceae. The genus *Russula* is easily morphologically distinguished from other common mushrooms by several recognizable features, such as free white gills, absence of partial veil or volva tissue on the stipe, and brittle fleshy basidiocarp due to the presence of spherocytes. However, given its high species diversity, *Russula* has been one of the most difficult genera for fungal systematics and taxonomy. The macromorphological features such as cap color can be highly variable within a species, and there are relatively few features to distinguish many closely related species within the genus. Much of the morphotypes found in the samples analyzed belong to the genus *Russula*, one of the most speciose genera in Africa, with about 200 species being identified in this continent, most of them edible, and with no reported serious toxicological problems [27]. Another important factor for the abundance of *Russula* in the dried mushroom mixtures sold may be the good drying and preservation capacity of the species in this genus.

DNA barcoding is a powerful tool for accurate identification at the species level, usually disentangling closely related and cryptic species that could not be distinguished by morphological characters. In this process, the selection of appropriate DNA barcodes is vital to assure resolution of species recognition, and even then, some fungi taxonomic groups can be quite challenging, as is the case of the *Russula* genus.

**Figure 3:**
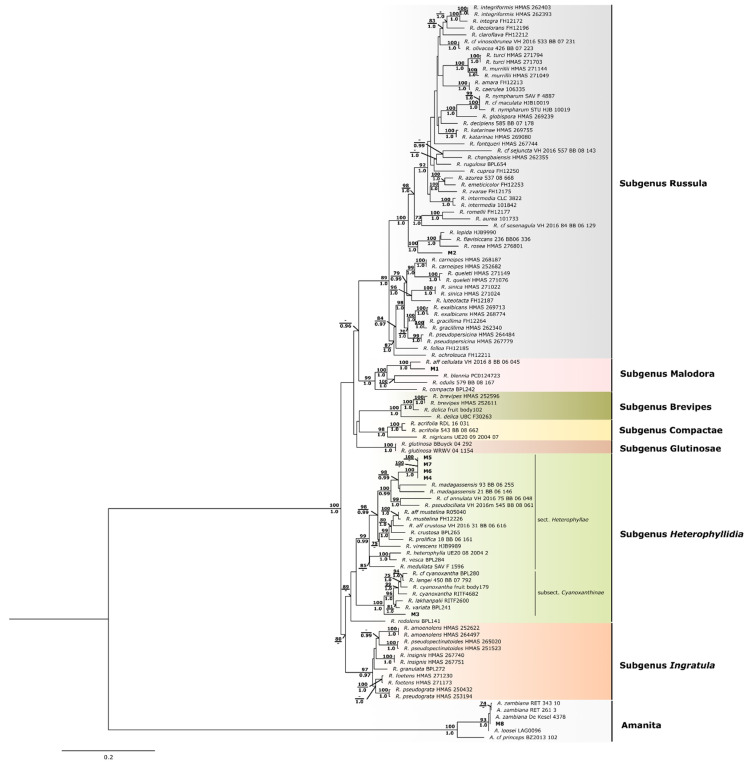
Maximum likelihood phylogenetic tree obtained from the concatenated dataset of ITS, LSU and rpb2 locus. ML Bootstrap support values and Bayesian posterior probability scores are given at the nodes (>70% and 0.95, respectively). The mushroom samples from Huila, Angola, numbered M1 to M8, are in bold.

In our study, we apply a sequencing multi-locus strategy based on the ITS region (ITS1-5.8S ribosomal RNA gene-ITS2), 28S large subunit ribosomal RNA gene (LSU), and RNA polymerase II second largest subunit (rbp2) gene to analyze our samples. Fragments ranging from 137–702 bp, 464–947 bp, and 691–805 were obtained for the ITS LSU and rbp2, respectively. Homology of identity was first investigated by querying our sequences against sequences available in the NCBI nucleotide database. Samples M1 to M7 had the most significant hits with sequences characterized as *Russula* (% of identity > 90%), but no species designation could be undoubtedly assigned. In contrast, a hit of 100% similarity was obtained for M8 sample sequences identified as belonging to the species *Amanita loosei* (synonym *A. zambiana*), in accordance with the morphological data. Having this into account, a phylogenetic analysis was performed using a large set of *Russula* and *Amanita* species sequences (Table S1; Figure 3).

Maximum likelihood and Bayesian Inference analyses from the concatenated dataset showed the existence of two major phylogenetic lineages that correspond to *Amanita* and *Russula* genera, as expected, placing M8 sample within *Amanita* species group (BS: 93 and BI:1.0) and the remaining samples (M1 to M7) within *Russula* clade (BS:100 and BI:1.0). Seven *Russula* subgenera as recognized by Buick et al. and Vera et al. [60,61] (*Russula*; *Malodora; Brevipes; Compactae; Glutinosae; Heterophyllidia* and *Ingratula*) were identified in our phylogeny. In this framework, M1 clustered within the subgenus Malodora (BS:99 and BI:1.0), showing a close relationship with *Russula cellulata* (BS:100 and BI:1.0). Given its phylogenetic placement, it is quite probable that sample M1 belongs to *R. cellulata*, which is an edible species common in central and southern Africa. M2 is in a basal position of a well-supported group (BS:100 and BI:1.0) that cluster *R. rosea* (synonym *R. lepida*) and *R. flavisiccans*, within subgenus Russula. However, despite the affinity to the *R. rosea*/*R. flavisiccans* group, no confident assignment can be made to a particular species. Similarly, M3 seems to be a basal lineage of a group identified as the subsection Cyanoxanthinae, section Heterophyllae of subgenus Heterophyllidia (BS:100 and BI:1.0) [62], but at this point, our data does not provide further specific resolution. The closest phylogenetic relationship of M3 is found with *R. cyanoxantha*, *R. variata*, *R. langei*, and *R. lakhanpalii*, species that are not reported for Africa. Subsection Cyanoxanthinae encompasses varied species characteristics and great similarities to *R. cyanoxantha*, as reported in a study for Central Africa [63] in which three new species similar to *R. cyanoxantha* were identified, namely *R. flavobrunnea*, *R. pseudopurpure*, and *R. striatoviridi*, being the first one recorded to Angola in miombo too. The remaining samples M4-M7 make up a well-supported, single, and differentiated sub-group (BS:100 and BI:1.0) within the subgenus Heterophyllidia. This result suggests that all these samples belong to the same species, although they exhibit morphological differences in the color of the hat (from yellow to red) and foot, in the size of the cystidia and basidia, as well as in the ornamentation of the spores. Although they cannot be assigned to any species with sequence resources publicly available, samples M4–M7 are closely related to *R. madagassensis*, common in Madagascar but not reported for the African continent. Considering the phylogeny, the possibility that these samples may belong to a species not yet described cannot be ruled out.

The limitations of distinguishing individual *Russula* species found in our work are not surprising as is well known that the genus *Russula* is one of the most highly diverse among Agaricomycetes, comprising over 780 species, and hard to resolve [23]. The high intra-specific variability and extensive phenotypic plasticity, as well as inaccurate descriptions in the literature and limited availability of DNA sequences in global databases, has long caused large difficulties in species resolution and taxonomic confusion [23,60]. As such, an increasing number of emerging species has arisen in recent years mainly based on molecular data, for which the combination of ITS-rpb2 has recently been suggested as the best target DNA barcode for *Russula* [23]. Our work using the barcode markers ITS, LSU, and rpb2 combined with morphological observations, despite limitations, allowed us to identify five different mushroom species in the dry samples of eight morphotypes from Humpata market (Huíla, Angola) (Table 1): *A. loosei*, *Russula aff. cellulata*, *Russula* sp1 (R. group rosea), *Russula* sp2 (subgenus Heterophyllidia, section Heterophyllae, subsection Cyanoxanthinae), and *Russula* sp3. (*R. aff. madagassensis*).

### 3.3. Chemical Characterization of Mushroom Species

The results accomplished for the nutritional composition and energetic value of the studied mushroom samples from Huila, Angola, are presented in Table 2. In all of the analyzes, carbohydrates were the most abundant macromolecule, followed by proteins, ashes, and fat. Among them, M1 (*Russula* aff. *cellulata*) and M2 (*Russula* sp1) presents the highest amount of carbohydrates (77.1 and 76.1 g/100 g dw, respectively), followed by M6, M4, M5 and M7 (*Russula* sp3), which present values ranging from 63.8 to 68.1 g/100 g dw, and finally M8 (*A. loosei*), which display the lowest value (61.7 g/100 g dw). As for proteins, similar amounts were observed between the studied mushrooms, varying from 14.1 to 16.2 g/100 g dw, except for M7, which presents the higher content (18.1 g/100 g dw), and M2 (12.64 g/100 g dw) the smallest. Additionally, very interesting amounts of ashes are detected in the analyzed samples, with values ranging from 6.85 to 16.19 g/100 g dw, suggestive of good micronutrient concentrations, which are involved in several mechanisms in the human organism. When compared to the above-mentioned constituents, the studied species are much less abundant in fat which, together with the high protein and carbohydrate contents, make them suitable for low-calorie diets. Although allegedly, samples M4 to M7 belong to the same species, they present significant differences in all parameters analyzed, which may possibly be related to the collection date of the mushrooms and their stage of development [64]. In a recent investigation, Kostić et al. [65] studied the nutritional composition of three *Russula* species from Serbia, among them *Russula rosea*, which molecular analysis showed affinity with our M2 sample. Overall, their results are partially consonant with ours, presenting similar amounts of carbohydrates (82.031 g/100 g dw) and proteins (12.21 g/100 g dw). However, the authors also detected much lower contents of ashes (4.9 g/100 g dw) and fat (0.84) when compared to our M2 sample, which may be due to the fact that these samples have different origins (Angola and Serbia), being therefore subject not only to different edaphoclimatic factors but also to different storage and processing techniques. About the other mushroom species under investigation and their possible affinities, to our best knowledge, this is the first study regarding their nutritional characterization.

The occurrence of free sugars was also analyzed in the mushrooms under investigation, and the results are described in Table 2. Mannitol and trehalose were the only two sugars identified in all samples, with mannitol presenting the highest concentration (4.88 to 20.48 g/100 g dw). Fructose was also detected in quite low amounts in M4 to M8 samples, ranging from 0.05 g/100 g dw found in *A. loosei* to 0.10–0.28 in *Russula* sp3. Through this analysis, it was possible to verify quite different values of the identified sugars, not only between species but within each species, which may be related, firstly, to genetic differences between species and, lastly, to possible different stages of development of the mushrooms as well as different processing/storage approaches [64]. The free sugar profile of *R. rosea* was also studied by Kostić et al. [65] whose results showed considerable discrepancies with our study since these authors only detected the presence of mannitol in this species and in amounts much higher (25.8 g/100 g dw) than those detected in the present study (16.49 g/100 g dw).

Regarding organic acids (Table 2), in all analyzed samples oxalic, quinic malic, citric, and fumaric acids were detected, with M6 (*Russula* sp3) presenting the highest content in total organic acids (11.80 g/100 g dw). As for samples M1, M2, M3, M6, M7 and M8, quinic acid is the main compounds (5.33, 5.65, 5.01, 4.72, 4.57 and 1.66 g/100 g dw, respectively). Regarding the total organic acids, *A. loosei* presents the lowest concentration among the analyzed mushrooms (3.86 g/100 g dw). Ribeiro et al. [64] studied the organic acids profile of *R. cyanoxantha*, which presents similarities with our M3 sample (*Russula* sp2). These authors were able to identify oxalic, citric, malic, quinic, and fumaric acids in this species, with malic and quinic acids presenting the highest concentration and oxalic the minor one, which was also observed in our study. In contrast, Kostić et al. [65] observed in *R. rosea* only the presence of oxalic and malic acids, and trace amounts of fumaric acid, which differs greatly from the present study, since quinic and citric acids were also identified, of which the first was present in greater amounts in this species. Given their ability to perform antioxidant activity, organic acids may play a protective role against a wide variety of diseases. Oxalic acid, for example, has proven to be able to perform antibacterial activity against a wide spectrum of microorganisms, whereas fumaric acid has been shown to have anti-inflammatory as well as antimicrobial properties, among others [66,67].

Lipophilic compounds, namely tocopherols and fatty acids were also examined in the studied mushroom samples (Table 2). It was possible to identify the presence of only α- and β-tocopherol isoforms, with the last being only identified in *Russula* sp1 in significant amounts (45.9 µg/100 g dw). Additionally, the α-tocopherol isoform was not detected in M4, M5, and M7 samples, although present in interesting amounts in the other analyzed samples, reaching its higher concentration in *A. loosei* (50.4 µg/100 g dw). Tocopherols were also able to perform antioxidant activity through the production of new free radicals and neutralization of existing ones. These compounds may consequently reduce the risk of degenerative diseases associated with oxidative stress [68,69].

The fatty acid assessment (Table 2) revealed oleic acid (C18:1n9c) as the main fatty acid present in all the analyzed samples, with values ranging from 42.81 to 55.80%, followed by palmitic acid (C16:0; 12.1 to 30.46%), and linoleic acid (C18:2n9c; 1.87 to 26.85%). Stearic acid (C18:0), in turn, was the one present in lower quantities. Comparing the studied mushrooms, *A. loosei* presents the highest content in oleic acid (55.80 ± 0.01%), *Russula* sp3 (M3) in palmitic acid (30.46%), and *Russula* sp1 (M2) in linoleic acid (26.85%). As about the percentage analysis of the saturated (SFA), monounsaturated (MUFA), and polyunsaturated (PUFA) fatty acids, the latter is present in smaller amounts in all samples under investigation, presenting its lowest value in M3 and M6 samples (2.2 and 11.5%, respectively), followed by SFAs, whose highest amount are present in the M3 sample (47.3%), and finally MUFA, the most prevalent class in overall samples (43.7 to 56.4%). The above-mentioned fatty acids were also detected by Kostić et al. [65] in *R. rosea* although, except for palmitic acid, in quite different amounts. Despite that, in this study, oleic acid was also the prevalent compound in *R. rosea*, as well as in our study, followed by linoleic acid. To the best of our knowledge, there are no available reports on the fatty acid profile of the other species in the study.

### 3.4. Determination of Phenolic Acids and Related Compounds, and Bioactive Properties

#### 3.4.1. Phenolic Acids and Related Compounds

The phenolic acids and related compounds assessment were performed in the hydroethanolic extracts of the mushrooms under investigation, with the attained results being presented in Table 3. In all experiments, protocatechuic, *p*-hydroxybenzoic, and *p*-coumaric acids, and a related compound, cinnamic acid, were identified. Overall, *p*-hydroxybenzoic acid was the main phenolic acid in all studied mushrooms, reaching its higher amount in *A. loosei* (320.1 µg/g extract) and *Russula* sp3, which samples present values ranging from 139.4 to 286.7 µg/g extract. Protocatechuic acid, in turn, presents its higher value in M2 sample (120 µg/g extract), and *p*-coumaric acid in the M8 sample (48.2 µg/g extract). As for cinnamic acid, its concentration is quite diverse between samples, with the minimum values being identified in M5 sample (3.43 µg/g extract), and the maximum in M7 (79 µg/g extract). In general, *Amanita loosei* presents the highest concentration (400.3) in phenolic acids (without cinnamic acid), followed by similar amounts in *Russula* sp3, namely in samples M4 and M5. The smallest concentration was, in turn, found in *Russula* sp2 (41 µg/g extract). Ribeiro et al. [70] in a study with entire mushrooms of *R. cyanoxantha*, were able to identify vestigial amounts of *p*-hydroxybenzoic acid, whereas in another study [64] this compound was not present in the same species, which may be due to the collection date and/or the development stages of the mushrooms. Additionally, Kostić et al. [65], when evaluating the phenolic profile o *R. rosea* methanolic and ethanolic extracts, were only capable to detect cinnamic acid (20 and 89 µg/g extract, respectively), which are present in lower amounts in our present study (16.5 µg/g extract). Moreover, in the present study, the above-mentioned compounds were all present in this species. Published data had shown that some bioactive properties performed by different mushroom species are related to their constitution in phenolic acids, namely anti-inflammatory activity by the expression of inflammatory markers, including the production of nitric oxide (NO), IL-6, and IL-1β [71].

#### 3.4.2. Antioxidant Activity

In the present investigation, the antioxidant capacity of the hydroethanolic extracts of mushroom samples was evaluated through lipid peroxidation inhibition, and the results for the thiobarbituric acid reactive substances (TBARS) assay are presented in Table 3. From the attained results, it was possible to observe that the highest lipid peroxidation was performed by M2, M7, and M4 samples (EC_50_ = 1.07, 1.08, and 1.15 mg/mL, respectively), whereas *Russula* sp2 demonstrated the lowest antioxidant capacity (EC_50_ = 2.47 mg/mL). Very few reports on the antioxidant capacity of the studied mushroom species are available in the literature. However, some of them describe a moderate capacity to perform this bioactivity by some of the species under investigation [50,64,72], which may be related to their content in organic acids, tocopherols, phenolic acids, and others, whose presence is associated with good antioxidant assets.

#### 3.4.3. Antimicrobial Activity

Ethanolic extracts of the mushroom samples under investigation presented antimicrobial activity against all tested strains at different degrees (Table 4). The minimum inhibitory concentration (MIC) values range between 0.5 and 4 mg/mL, whereas the minimum bactericidal concentration (MBC) was between 1 and 8 mg/mL. The most sensitive strains analysed were *E. coli* (MICs = 0.5 with all extracts), and *B. cereus* (MICs 0.5–2 mg/mL), whereas the most resilient was *S. aureus* (MICs 1–4 mg/mL). None of the extracts investigated revealed greater antibacterial activity than the commercial antibiotics used as a positive control, namely streptomycin and ampicillin. The antibacterial potential of *R. rosea* ethanolic and methanolic extracts were also studied by Kostić et al. [52], whose results also demonstrated considerable growth inhibition of the analyzed strains., with the best MIC values being accomplished by the methanolic extract against *M. luteus* and by the ethanolic one against *S. dysgalactiae* (MIC = 0.20 mg/mL, both).

The antifungal activity of the tested mushroom’s hydroethanolic extracts resulted in MIC and MFC (minimum fungicidal concentration) values slightly above those of positive controls, ketoconazole, and bifonazole (Table 5). Despite that, all extracts showed good antifungal capacity, mainly against *T. viride* (MICs 0.5 mg/mL), and to a lesser extent against *Penicillium verrucosum* var. *cyclopium* (MICs 1–4 mg/mL). To the best of our knowledge, this is the first report on the antifungal activity of the species under investigation.

### 3.5. Multivariate Analysis of Chemical and Functional Characteristics of Samples

Projecting the chemical and functional variables analyzed in a PCA revealed a close correspondence between the chemical and functional properties of the samples, distinguishing completely the eight morphotypes. Interestingly, the cluster organization obtained corresponded directly with the five taxa identified by morphological and molecular characterization. Additionally, considering the common names registered in Huila, a good correspondence between the local names, the taxonomic entities, and their chemical and functional properties can be noted (Figure S1).

There is important traditional knowledge about the identification of WEMs as well as their properties. Regarding local common names, it is interesting to note that the names given to the morphotypes have a good correspondence with nutritional characteristics, as is the case of *chelenes* (yellow, black, and red) that in the multivariate analysis were grouped together (Figure S1). This wealth of traditional knowledge about natural resources, which is learned and transmitted orally, may be eroding and must be preserved and documented.

## 4. Conclusions

Despite the great socio-economic importance and the potential to contribute to food security for both rural and urban populations, very little information is available so far on the edible mushroom species in Angola and their food and functional properties. However, although many African mushroom species are edible and have good nutritional properties, there is also a traditional belief that eating wild mushrooms can be dangerous and even deadly. Therefore, the correct identification of mushroom species and the research about their properties are important for the sustainable and safe use of this renewable natural resource. The present study represents the first assessment of wild edible mushrooms consumed and sold in local markets in Angola, providing a comprehensive profiling at the morphological, molecular, chemical, nutritional, and bioactive levels. Although species identification proves to be very difficult, our results brought useful insights that can assist future studies aiming to improve the mushroom taxonomic classification.

For the majority of the analyzed mushroom species, nutritional, chemical, antioxidant, and antimicrobial activities are reported for the first time, indicating that these are valuable sources of nutrients and bioactive compounds, with good in vitro performances. Given its rich composition in a wide range of compounds of interest, and the scarcity of studies focusing on these species, investigations must go further to explore them as nutraceuticals and in the development of new value-added food products. Additionally, the bioactive properties displayed by mushrooms must be fully understood and explored in the future, both through in vitro and in vivo studies.

## Figures and Tables

**Table 1:** Characterization of the eight morphotypes found in the dried mushrooms sample from Humpata market, Angola (Note: cap and stipe dimensions were taken on dried specimens).

Samples	Species	Common Name	Ecology/Morphology
M1	*Russula aff*. *cellulata* Buyck	Ombando (Umbundu)	Occurs in closed to open Miombo woodland, under the tree canopy, sometimes in moss-covered soil, mainly from March to May. Cap: brown cream or light brown, often cracked, 5–10 cm Ø. Stipe: whitish, 4–5 × 1.5–2.5 cm. Spores: hyaline, with small warts, very occasionally connected by fine lines, subglobolose 6.5–8 μm. Basidia: 50 × 10 μm.
M2	*Russula* sp1 [*R.* gr. *rosea* Pers. (=*R. lepida* Fr.)]	Opembe (Nyaneka); Membe, Umputu (Umbundu)	Occurs in closed to open Miombo woodland, under the tree canopy, often near the base of the tree trunks, mainly in March to May. Cap: vinaceous or blood-red, 3.5–7.5 cm Ø. Stipe: white with red or rosaceous flush, 3–5 × 1.8–2 cm. Spores: with warts, some connected by fine lines, broadly ellipsoid, 10 × 9 μm. Basidia: 45–50 × 10 μm.
M3	*Russula* sp2 (subgenus Heterophyllidia, section Heterophyllae, subsection Cyanoxanthinae)	Chelene preto [black chelene] (Umbundu/Nyaneka)	Occurs in closed to open Miombo woodland, under the tree canopy, mainly in March to May. Cap: dark purple with greenish tones, mucilaginous, 3–5 cm Ø. Stipe: whitish, 3.5–4 × 2 cm. Spores: with warts, ellipsoid 7 × 5.5 μm. Basidia: 9 × 5 μm.
M4	*Russula* sp3. (*R.* aff *madagassensis* R. Heim)	Chelene amarelo [yellow chelene] (Umbundu/Nyaneka)	Occurs in open Miombo woodland and open rocky areas on sloping terrains, mainly in March to May. Cap: brownish yellow, mucilaginous, 5–6 cm Ø. Stipe: 3.5–4 × 1.5–2 cm. Spores: with warts, ellipsoid, 7–8 × 5 μm. Basidia: 55 × 10 μm.
M5	*Russula* sp3. (*R.* aff *madagassensis* R. Heim)	Chelene amarelo [yellow chelene] (Umbundu/Nyaneka)	Occurs in open Miombo woodland and open rocky areas on sloping terrains, mainly in March to May. Cap: brownish yellow, viscid, 5–6 cm Ø. Stipe: 3.5–4 × 1.5–2 cm. Spores: sub-spherical, with small warts interconnected by narrow veins giving a reticulated aspect, 7–7.5 μm. Basidia: 50–55.5 × 12 μm.
M6	*Russula* sp3. (*R.* aff *madagassensis* R. Heim)	Chelene vermelho [red chelene] (Umbundu/Nyaneka)	Occurs in open Miombo woodland, under the tree canopy, mainly in March to May. Cap: reddish or pink 5–6 cm Ø. Stipe: 3.5–4 × 1.5–2 cm. Spores: ornamented with small warts interconnected by narrow veins giving a reticulated aspect, spherical, 8–9 μm. Basidia: 50 × 10 μm.
M7	*Russula* sp3. (*R.* aff *madagassensis* R. Heim)	Chelene vermelho [red chelene] (Umbundu/Nyaneka)	Occurs in open Miombo woodland, under the tree canopy, mainly in March to May. Cap: reddish or light pink, 5–6 cm Ø; Stipe: 3.5–4 × 1.5–2 cm. Spores: ornamented with small warts interconnected by narrow veins giving a reticulated aspect, spherical, 8–9 μm. Basidia: 50 × 10–12.5 μm.
M8	*Amanita loosei* Beeli (=*A. zambiana*)	Ndenda (Umbundu); Ondenda (Nyaneka)	Occurs in closed to open Miombo woodland, under the tree canopy, often in rocky soil, mainly in December to May. Cap: brownish at the centre becoming paler towards the margin, 10–25 cm Ø, (can reach 30–40 cm in fresh). Stipe: white, 5–10 × 2–3 cm. Ring and volva: present. Spores: hyaline, smooth, spherical a sub-spherical, 12 μm. Basidia: 50 × 10 μm

**Table 2:** Nutritional, hydrophilic, and lipophilic compounds of the studied mushrooms’ samples from Huila, Angola (mean ± SD, *n* = 3).

	M1 (*Russula* aff. *cellulata*)	M2 (*Russula* sp1)	M3 (*Russula* sp2)	M4 (*Russula* sp3)	M5 (*Russula* sp3)	M6 (*Russula* sp3)	M7 (*Russula* sp3)	M8 *(Amanita loosei)*
Nutritional value (g/100 g dw)								
Fat	1.99 ± 0.05 ^h^	2.17 ± 0.06 ^g^	5.24 ± 0.02 ^b^	2.43 ± 0.02 ^f^	3.70 ± 0.06 ^c^	3.16 ± 0.01 ^e^	3.51 ± 0.09 ^d^	8.20 ± 0.01 ^a^
Proteins	14.1 ± 0.5 ^d^	12.6 ± 0.4 ^e^	14.1 ± 0.1 ^d^	15.8 ± 0.1 ^bc^	16.2 ± 0.1 ^ab^	15.4 ± 0.1 ^c^	18.1 ± 0.3 ^a^	15.2 ± 0.2 ^c^
Ash	6.85 ± 0.03 ^f^	9.20 ± 0.01 ^e^	15.87 ± 0.01 ^a^	14.47 ± 0.03 ^b^	14.05 ± 0.01 ^c^	13.39 ± 0.04 ^d^	16.19 ± 0.04 ^a^	14.92 ± 0.06 ^b^
Carbohydrates	77.1 ± 0.4 ^a^	76.1 ± 0.4 ^b^	64.82 ± 0.05 ^f^	67.3 ± 0.1 ^d^	66.0 ± 0.1 ^e^	68.1 ± 0.1 ^c^	63.8 ± 1.4 ^f^	61.7 ± 0.1 ^g^
Energy value (kcal/100 g dw)	382.6 ± 0.3 ^a^	374.1 ± 0.2 ^b^	362.7 ± 0.1 ^c^	354.3 ± 0.2 ^d^	362.3 ± 0.2 ^c^	362.2 ± 0.1 ^c^	352.8 ± 0.4 ^d^	381.4 ± 0.2 ^a^
Hydrophilic compounds (free sugars and organic acids, g/100 g dw)								
Frutose	nd	nd	nd	0.16 ± 0.03 ^c^	0.10 ± 0.01 ^d^	0.23 ± 0.01 ^b^	0.28 ± 0.04 ^a^	0.05 ± 0.01 ^e^
Mannitol	20.48 ± 0.04 ^a^	16.49 ± 0.07 ^b^	12.45 ± 0.03 ^f^	13.67 ± 0.03 ^d^	14.29 ± 0.01 ^c^	13.01 ± 0.04 ^e^	6.71 ± 0.02 ^g^	4.88 ± 0.07 ^h^
Trehalose	0.77 ± 0.03 ^g^	0.83 ± 0.01 ^e^	1.28 ± 0.04 ^c^	1.38 ± 0.02 ^b^	0.71 ± 0.05 ^h^	1.08 ± 0.08 ^d^	0.77 ± 0.07 ^f^	6.50 ± 0.08 ^a^
Total sugars	21.25 ± 0.06 ^a^	17.32 ± 0.07 ^b^	13.74 ± 0.01 ^f^	15.21 ± 0.02 ^c^	15.09 ± 0.06 ^d^	14.32 ± 0.04 ^e^	7.76 ± 0.09 ^h^	11.43 ± 0.01 ^g^
Oxalic	0.022 ± 0.001 ^e^	0.029 ± 0.002 ^e^	0.105 ± 0.001 ^c^	0.115 ± 0.003 ^c^	0.106 ± 0.001 ^c^	0.49 ± 0.02 ^a^	0.150 ± 0.003 ^b^	0.057 ± 0.001 ^d^
Quinic	5.33 ± 0.05 ^b^	5.65 ± 0.06 ^a^	5.01 ± 0.01 ^c^	2.59 ± 0.01 ^f^	2.48 ± 0.01 ^g^	4.72 ± 0.01 ^d^	4.57 ± 0.01 ^e^	1.66 ± 0.01 ^h^
Malic	2.39 ± 0.05 ^f^	1.64 ± 0.01 ^g^	3.99 ± 0.01 ^d^	4.09 ± 0.03 ^c^	3.48 ± 0.01 ^e^	4.55 ± 0.01 ^a^	4.39 ± 0.02 ^b^	1.42 ± 0.01 ^h^
Citric	0.85 ± 0.01 ^f^	0.84 ± 0.01 ^f^	1.22 ± 0.01 ^e^	1.71 ± 0.01 ^c^	1.56 ± 0.01 ^d^	2.04 ± 0.01 ^b^	2.60 ± 0.06 ^a^	0.718 ± 0.007 ^g^
Fumaric	tr	tr	tr	tr	tr	tr	tr	tr
Total organic acids	8.59 ± 0.01 ^d^	8.16 ± 0.05 ^f^	10.34 ± 0.01 ^c^	8.51 ± 0.02 ^e^	7.63 ± 0.01 ^g^	11.80 ± 0.01 ^a^	11.71 ± 0.08 ^b^	3.86 ± 0.01 ^h^
Lipophilic compounds (fatty acids, % and tocopherol, µg/100 g dw)								
C16:0	19.81 ± 0.03 ^d^	12.1 ± 0.1 ^e^	30.46 ± 0.02 ^a^	23.2 ± 0.3 ^c^	20.03 ± 0.04 ^d^	24.5 ± 0.1 ^b^	19.95 ± 0.06 ^d^	19.5 ± 0.1 ^d^
C18:0	5.03 ± 0.04 ^g^	9.4 ± 0.1 ^b^	11.9 ± 0.1 ^a^	6.68 ± 0.09 ^d^	5.93 ± 0.01 ^e^	7.78 ± 0.1 ^c^	6.56 ± 0.01 ^d^	5.15 ± 0.05 ^f^
C18:1n9c	50.8 ± 0.1 ^c^	48.55 ± 0.05 ^d^	46.67 ± 0.08 ^e^	42.810.5 ^f^	48.9 ± 0.5 ^d^	53.3 ± 0.01 ^b^	50.3 ± 0.3 ^c^	55.80 ± 0.01 ^a^
C18:2n6c	17.4 ± 0.1 ^e^	26.85 ± 0.07 ^a^	1.87 ± 0.01 ^h^	19.04 ± 0.09 ^c^	20.7 ± 0.1 ^b^	8.6 ± 0.1 ^g^	18.7 ± 0.1 ^c,d^	15.7 ± 0.2 ^f^
SFA (%)	26.4 ± 0.1 ^f^	22.2 ± 0.1 ^g^	47.3 ± 0.1 ^a^	35.0 ± 0.5 ^b^	27.4 ± 0.7 ^e^	34.5 ± 0.1 ^c^	28.3 ± 0.1 ^d^	26.22 ± 0.1 ^f^
MUFA (%)	51.8 ± 0.1 ^c^	49.1 ± 0.1 ^g^	50.6 ± 0.1 ^e^	43.7 ± 0.6 ^h^	49.7 ± 0.5 ^f^	54.0 ± 0.1 ^b^	51.0 ± 0.3 ^d^	56.4 ± 0.1 ^a^
PUFA (%)	21.8 ± 0.1 ^c^	28.7 ± 0.2 ^a^	2.2 ± 0.1 ^h^	21.3 ± 0.1 ^d^	22.9 ± 0.1 ^b^	11.5 ± 0.1 ^g^	20.7 ± 0.2 ^e^	17.4 ± 0.1 ^f^
α-Tocopherol	36.9 ± 0.7 ^b^	22.8 ± 0.8 ^d^	23.1 ± 0.1 ^c^	nd	nd	21.8 ± 0.8 ^e^	nd	50.4 ± 0.8 ^a^
β-Tocopherol	nd	45.9 ± 0.4	nd	nd	nd	nd	nd	nd
Total tocopherols	36.9 ± 0.7 ^c^	68.7 ± 1.3 ^a^	23.1 ± 0.1 ^d^	-	-	21.8 ± 0.8 ^e^	-	50.4 ± 0.8 ^b^

nd—not detected, tr—traces; different letters in the same line show significant difference between means according to Tukey’s HSD test (*p* < 0.05); C16:0—palmitic acid; C18:0—stearic acid; C18:1n9—oleic acid; C18:2n6—linoleic acid; SFA—Saturated fatty acids; MUFA—Monounsaturated fatty acids; PUFA—Polyunsaturated fatty acids.

**Table 3:** Antioxidant activity, phenolic acids, and related compounds of the studied mushroom hydroethanolic extracts (mean ± SD, *n* = 3).

Morphotypes/Species	M1 (*Russula* aff. *cellulata*)	M2 (*Russula* sp1)	M3 (*Russula* sp2)	M4 (*Russula* sp3)	M5 (*Russula* sp3)	M6 (*Russula* sp3)	M7 (*Russula* sp3)	M8 *(Amanita loosei)*
Antioxidant activity (EC_50_ mg/mL)								
TBARS	2.09 ± 0.05 ^c^	1.07 ± 0.08 ^e^	2.47 ± 0.04 ^a^	1.15 ± 0.03 ^d^	2.25 ± 0.06 ^b^	2.52 ± 0.05 ^a^	1.08 ± 0.09 ^e^	2.25 ± 0.08 ^b^
Phenolic acids and related compounds (µg/g of extract)								
Protocatechuic acid	9.1 ± 0.5 ^g^	120 ± 1 ^a^	2.89 ± 0.01 ^h^	56.7 ± 0.6 ^b^	46.3 ± 0.3 ^c^	33.5 ± 0.4 ^d^	19.8 ± 0.4 ^f^	31.9 ± 0.6 ^e^
*p*-Hydroxybenzoic acid	93.3 ± 0.6 ^f^	28.0 ± 0.4 ^h^	31.8 ± 0.9 ^g^	274.0 ± 0.1 ^c^	286.7 ± 0.2 ^b^	145.2 ± 0.5 ^d^	139.4 ± 0.9 ^e^	320.1 ± 0.4 ^a^
*p*-Coumaric acid	21.4 ± 0.3 ^f^	15 ± 1 ^g^	6.7 ± 0.2 ^h^	43.6 ± 0.4 ^c^	45.1 ± 0.5 ^b^	39.4 ± 0.6 ^d^	29.3 ± 0.6 ^e^	48.2 ± 0.5 ^a^
Total	123.8 ± 0.5 ^g^	163.3 ± 0.8 ^f^	41 ± 1 ^h^	374 ± 1 ^c^	378.1 ± 0.9 ^b^	218.1 ± 0.1 ^d^	188 ± 1 ^e^	400.3 ± 0.6 ^a^
Cinnamic acid	55.0 ± 0.7 ^b^	16.5 ± 0.3 ^c^	6.37 ± 0.07 ^d^	6.2 ± 0.1 ^d^	3.43 ± 0.01 ^e^	3.8 ± 0.7 ^e^	79 ± 3 ^a^	16.3 ± 0.9 ^c^

EC_50_: extract concentration corresponding to a 50% of antioxidant activity. Trolox EC_50_ values: 23 µg/mL (TBARS inhibition) and 19.6 µg/mL; Different letters in the same line show significant difference between means according to Tukey’s HSD test (*p* < 0.05).

**Table 4:** Antibacterial activity of the studied mushroom hydroethanolic extracts (mg/mL).

		*Staphylococcus aureus* (ATCC 11632)	*Bacillus cereus* (Clinical Isolate)	*Listeria monocytogenes* (NCTC 7973)	*Escherichia coli* (ATCC 25922)	*Salmonella typhimurium* (ATCC 13311)	*Enterobacter cloacae* (ATCC 35030)
M1 (*Russula* aff. *cellulata*)	MIC	2	1	2	0.5	1	2
	MBC	4	2	4	1	2	4
M2 (*Russula* sp1)	MIC	1	1	2	0.5	1	1
	MBC	2	2	4	1	2	2
M3 (*Russula* sp2	MIC	2	1	2	0.5	1	1
	MBC	4	2	4	1	2	2
M4 (*Russula* sp3)	MIC	1	0.5	1	0.5	1	2
	MBC	2	1	2	1	2	4
M5 (*Russula* sp3)	MIC	4	1	2	0.5	1	2
	MBC	8	2	4	1	2	4
M6 (*Russula* sp3)	MIC	1	0.5	2	0.5	2	2
	MBC	2	1	4	1	4	4
M7 (*Russula* sp3)	MIC	4	1	1	0.5	1	2
	MBC	8	2	2	1	2	4
M8 (*Amanita loosei*)	MIC	1	1	2	0.5	1	2
	MBC	2	2	4	1	2	4
Streptomycin	MIC	0.10	0.04	0.20	0.20	0.20	0.25
	MBC	0.20	0.10	0.30	0.30	0.30	0.50
Ampicilin	MIC	0.25	0.25	0.40	0.40	0.25	0.75
	MBC	0.40	0.45	0.50	0.50	0.50	1.20

MIC—minimal inhibitory concentration; MBC—minimal bactericidal concentration.

**Table 5:** Antifungal activity of studied mushrooms hydroethanolic extracts (mg/mL).

		*Aspergillus fumigatus* (Human Isolate)	*Aspergillus niger* (ATCC 6275)	*Aspergillus versicolor* (ATCC11730)	*Penicillium funiculosum* (ATCC 36839)	*Trichoderma viride* (IAM 5061)	*Penicillium verrucosum var. cyclopium* (Food Isolate)
M1 (*Russula* aff. *cellulata*)	MIC	1	1	1	2	0.5	4
	MFC	2	2	2	4	1	8
M2 (*Russula* sp1)	MIC	0.5	1	1	1	0.5	1
	MFC	1	2	2	2	1	2
M3 (*Russula* sp2	MIC	0.5	1	1	0.5	0.5	1
	MFC	1	2	2	1	1	2
M4 (*Russula* sp3)	MIC	0.5	2	1	0.5	0.5	1
	MFC	1	4	2	1	1	2
M5 (*Russula* sp3)	MIC	0.5	1	1	0.5	0.5	1
	MFC	1	2	2	1	1	2
M6 (*Russula* sp3)	MIC	0.5	1	1	1	0.5	4
	MFC	1	2	2	2	1	8
M7 (*Russula* sp3)	MIC	0.5	1	1	0.5	0.5	1
	MFC	1	2	2	1	1	2
M8 (*Amanita loosei*)	MIC	1	1	1	1	0.5	1
	MFC	2	2	2	2	1	2
Ketoconazole	MIC	0.25	0.20	0.20	0.20	2.50	0.20
	MFC	0.50	0.50	0.50	0.50	3.50	0.30
Bifonazole	MIC	0.15	0.15	0.10	0.20	0.20	0.10
	MFC	0.20	0.20	0.20	0.25	0.25	0.20

MIC—minimal inhibitory concentration; MFBC—minimal fungicidal concentration.

## Data Availability

The data used in the preparation of the article are presented in the published version and Supplementary Table S1. The DNA sequences are deposited in GenBank (accession numbers in Table S1).

## Supplementary Materials

The following supporting information can be downloaded at: https://www.mdpi.com/article/10.3390/foods11203240/s1, Table S1: List of specimens and respective accession numbers for the DNA sequences used in this study [23,73,74,75,76,77,78]. Figure S1: Principal Component Analysis (PCA) plot obtained with the nutritional and functional chemistry variables from Table 2, Table 3, Table 4 and Table 5. Samples are marked with different symbols according to phylogenetic groups (Figure 3), and the local common names are included
