# Supplementary material for: Biochemical and Molecular Profiling of Wild Edible Mushrooms from Huila, Angola
Source: Foods. 2022 Oct 17;11(20):3240. doi: 10.3390/foods11203240 (PMC9601281; doi:10.3390/foods11203240)
Supplement: Supplementary file 1 [file foods-11-03240-s001.zip › foods-1941359-supplementary.pdf]

foods

MDPI

Supplementary Materials

Table S1. List of specimens and respective accession numbers for the DNA sequences used in this study.

Species	Voucher/isolate ID	Origin	ITS	LSU28S	rpb2	Reference
**AMANITACEAE**						
Amanita cf. princeps	BZ2013 102	Thailand	MF461588	--	MF440408	[75]
Amanita loosii	LAG0096	--	MW829789	MK908831	--	Direct Submission NCBI
Amanita zambiana	RET 343-10	Zambia	--	KF877312	KF877097	[76]
Amanita zambiana	RET 261-3	Burundi	--	KF877311	KF877096	[76]
Amanita zambiana	De Kesel 4378	Togo	--	KF877309	KF877095	[76]
Amanita loosei	M8	Angola	OP082440	OP082425	OP099864	This study
**RUSSULA**						
Russula acrifolia	RDL 16-031	Italy (Tuscany)	MW172313	MW182474	MW306679	[77]
Russula acrifolia	543/BB 08.662	--	--	KU237535	KU237821	Direct Submission NCBI
Russula aff. *cellulata*	VH 2016/8/BB 06.045	--	--	KU237454	KU237740	Direct Submission NCBI
Russula aff. *crustosa*	VH 2016/31/BB 06.616	--	--	KU237461	KU237747	Direct Submission NCBI
Russula aff. *mustelina*	r-05040	USA (California)	JF834362	JF834509	JF834459	Direct Submission NCBI
Russula amoenolens	HMAS 264497	China	KX441078	KX441325	KX442066	Direct Submission NCBI
Russula amoenolens	HMAS 252622	China	KX441035	KX441282	KX442023	Direct Submission NCBI
Russula amara	FH-12-213	--	KT933998	KT933859	KT933930	[78]
Russula aurea	101733	Estonia	--	KX812878	KX813659	Direct Submission NCBI
Russula azurea	537/08.668	--	JN944002	JN940591	--	Direct Submission NCBI
Russula blennia	PC0124723	--	MH545687	KU237556=NG_064398	KU237842	Direct Submission NCBI
Russula brevipes	HMAS 252596	China	KX441030	KX441277	KX442018	Direct Submission NCBI
Russula brevipes	HMAS 252611	China	KX441033	KX441280	KX442021	Direct Submission NCBI
Russula carneipes	HMAS 252682	China	KX441039	KX441286	KX442027	Direct Submission NCBI
Russula carneipes	HMAS 268187	China	KX441116	KX441363	KX442104	Direct Submission NCBI
Russula changbaiensis	HMAS 262355	China	KX441057	KX441304	KX442045	Direct Submission NCBI
Russula caerulea	106335	--	--	KX812895	KX813676	Direct Submission NCBI
Russula cf. *annulata*	VH 2016/75/BB 06.048	--	--	KU237470	KU237756	Direct Submission NCBI
Russula cf. *cyanoxantha*	BPL280	USA (Tennessee)	KT933976	KT933837	--	[78]
Russula cf. *sesenagula*	VH 2016/84/BB 06.129	--	--	KU237473	KU237759	Direct Submission NCBI

Russula cf. *vinosobrunea*	VH 2016/533/BB 07.231	--	--	KU237525	KU237811	Direct Submission NCBI
Russula claroflava	FH-12-212	Germany	KT933997	KT933858	KT933929	[78]
Russula compacta	BPL242	--	KT933960	KT933819	KT933890	[78]
Russula crustosa	BPL265	--	KT933966	KT933826	KT933898	[78]
Russula cuprea	FH12250	--	KT934010	KT933871	KT933942	[78]
Russula cyanoxantha	RITF4682	China	MW646981	MW646993	--	[79]
Russula cyanoxantha	fruit body179	China	MN704834	MN710574	--	Direct Submission NCBI
Russula decipiens	585/BB 07.178	--	--	KU237569	KU237855	Direct Submission NCBI
Russula decolorans	FH12196	--	KT933992	KT933853	KT933924	[78]
Russula delica	fruit body102	--	MN704836	MN710576	--	Direct Submission NCBI
Russula delica	UBC:F30263	--	KX812842	KX812864	--	Direct Submission NCBI
Russula edulis	strain 579/BB 08.167	--	--	KU237564	KU237850	Direct Submission NCBI
Russula emeticicolor	FH12253	--	KT934011	KT933872	KT933943	[78]
Russula exalbicans	HMAS 268774	China (Sichuan)	MG493205	MG493219	MG495110	[80]
Russula exalbicans	HMAS 269713	China (Sichuan)	KX441161	KX441408	KX442149	Direct Submission NCBI
Russula flavisiccans	strain 236/BB 06.336	--	--	KU237485	KU237771	Direct Submission NCBI
Russula fellea	FH12-185	Germany	KT933989	KT933850	KT933921	[78]
Russula foetens	HMAS 271173	China	KX441223	KX441470	KX442211	Direct Submission NCBI
Russula foetens	HMAS 271230	China	KX441229	KX441476	KX442217	Direct Submission NCBI
Russula fontqueri	HMAS 267744	China	KX441096	KX441343	KX442084	Direct Submission NCBI
Russula glutinosa	WRWV 04-1154	USA (west Virginia)	MN315540	MN315511	MN326798	[81]
Russula glutinosa	B.Buyck 04-292	USA (North Carolina)	MN315543	MN315512	MN326795	[81]
Russula granulata	BPL272	USA (Tennessee)	KT933971	KT933832	KT933903	[78]
Russula globispora	HMAS 269239	China	KX441136	KX441383	KX442124	Direct Submission NCBI
Russula gracillima	FH12-264	Germany	KR364094	KR364226	KR364342	[78]
Russula gracillima	HMAS 262340	China	MG493206	MG493221	MG495112	[80]
Russula heterophylla	UE20.08.2004-2	Sweden	DQ422006	--	DQ421951	Direct Submission NCBI
Russula intermedia	CLC 3822	USA (Alaska)	MT583250	--	MT500711	Direct Submission NCBI
Russula intermedia	101842	Estonia	--	KX812888	KX813669	Direct Submission NCBI
Russula insignis	HMAS 267740	China	KX441094	KX441341	KX442082	Direct Submission NCBI
Russula insignis	HMAS 267751	China	KX441099	KX441346	KX442087	Direct Submission NCBI
Russula integra	FH12172	Germany	KT933984	KT933845	KT933916	[78]
Russula integriformis	HMAS 262393	China	KX441065	KX441312	KX442053	Direct Submission NCBI

Russula integriformis	HMAS 262403	China	KX441066	KX441313	KX442054	Direct Submission NCBI
Russula katarinae	HMAS 269080	China	KX441133	KX441380	KX442121	Direct Submission NCBI
Russula katarinae	HMAS 269755	China	KX441163	KX441410	KX442151	Direct Submission NCBI
Russula langei	450/BB 07.792	--	--	KU237510	KU237796	Direct Submission NCBI
Russula lepida	HJB9990	--	DQ422013	--	DQ421954	Direct Submission NCBI
Russula luteotacta	FH12187	--	KT933991	KT933852	KT933923	[78]
Russula maculata	HJB10019	Belgium	DQ422015	--	DQ421956	Direct Submission NCBI
Russula madagassensis	strain 21/BB 06.146	--	--	KU237456	KU237742	Direct Submission NCBI
Russula madagassensis	strain 93/BB 06.255	--	--	KU237475	KU237761	Direct Submission NCBI
Russula medullata	SAV F-1596	Slovakia	MT738281	MT738257	--	Direct Submission NCBI
Russula mustelina	FH12226	--	KT934005	KT933866	KT933937	[78]
Russula murrillii	HMAS 271049	China	KX441191	KX441438	KX442179	Direct Submission NCBI
Russula murrillii	HMAS 271144	China	KX441213	KX441460	KX442201	Direct Submission NCBI
Russula nigricans	UPS UE20.09.2004–07	--	DQ422010	DQ422010	DQ421952	Direct Submission NCBI
Russula nympharum	STU (HJB 10019)	--	--	MG944282	MG944258	Direct Submission NCBI
Russula nympharum	SAV F-4887	--	MG948640	--	MG944260	Direct Submission NCBI
Russula olivacea	426/BB 07.223	--	--	KU237492	KU237778	Direct Submission NCBI
Russula ochroleuca	FH12-211	--	KT933996	KT933857	KT933928	[78]
Russula prolifica	18/BB 06.161	--	--	KU237455	KU237741	Direct Submission NCBI
Russula pseudociliata	VH 2016m/545/BB08.061	--	MH545688	KU237537	KU237823	Direct Submission NCBI
Russula pseudograta	HMAS 250432	China	KX441012	KX441259	KX442000	Direct Submission NCBI
Russula pseudograta	HMAS 253194	China	KX441049	KX441296	KX442037	Direct Submission NCBI
Russula pseudopectinatoides	HMAS 251523	China	KX441016	KX441263	KX442004	Direct Submission NCBI
Russula pseudopectinatoides	HMAS 265020	China	KX441089	KX441336	KX442077	Direct Submission NCBI
Russula pseudopersicina	HMAS 264484	China	KX441077	KX441324	KX442065	Direct Submission NCBI
Russula pseudopersicina	HMAS 267779	China	KX441105	KX441352	KX442093	Direct Submission NCBI
Russula queleti	HMAS 271076	China	MG493211	MG493226	MG495117	[80]
Russula queleti	HMAS 271149	China	KX441215	KX441462	KX442203	Direct Submission NCBI
Russula redolens	BPL141	--	KT933950	KT933808	KT933879	[78]
Russula romellii	FH12177	--	KT933987	KT933848	KT933919	[78]

Russula rugulosa	BPL654	--	KY509494	--	KY701373	[78]
Russula rosea	HMAS 276801	China	LT602969	LT602946	KX442557	Direct Submission NCBI
Russula sinica	HMAS 271022	China	KX441186	KX441433	KX442174	Direct Submission NCBI
Russula sinica	HMAS 271024	--	KX441187	KX441434	KX442175	Direct Submission NCBI
Russula cf. *sejuncta*	VH 2016/557/BB 08.143	--	--	KU237547	KU237833	Direct Submission NCBI
Russula turci	HMAS 271703	China	KX441237	KX441484	KX442225	Direct Submission NCBI
Russula turci	HMAS 271794	China	KX441246	KX441493	KX442234	Direct Submission NCBI
Russula variata	BPL241	--	KT933959	KT933818	KT933889	[78]
Russula vesca	BPL284	--	KT933978	KT933839	KT933910	[78]
Russula virescens	HJB9989	Belgium	DQ422014	--	DQ421955	Direct Submission NCBI
Russula zvarae	FH12-175	Germany	KT933986	KT933847	KT933918	[78]
Russula aff. *cellulata*	M1	Angola	OP082434	OP082418	OP099858	This study
Russula sp. 1	M2	Angola	--	OP082419	OP099859	This study
Russula sp. 2	M3	Angola	OP082435	OP082420	OP099860	This study
Russula sp. 3	M4	Angola	OP082436	OP082421	--	This study
Russula sp. 3	M5	Angola	OP082437	OP082422	OP099861	This study
Russula sp. 3	M6	Angola	OP082438	OP082423	OP099862	This study
Russula sp. 3	M7	Angola	OP082439	OP082424	OP099863	This study

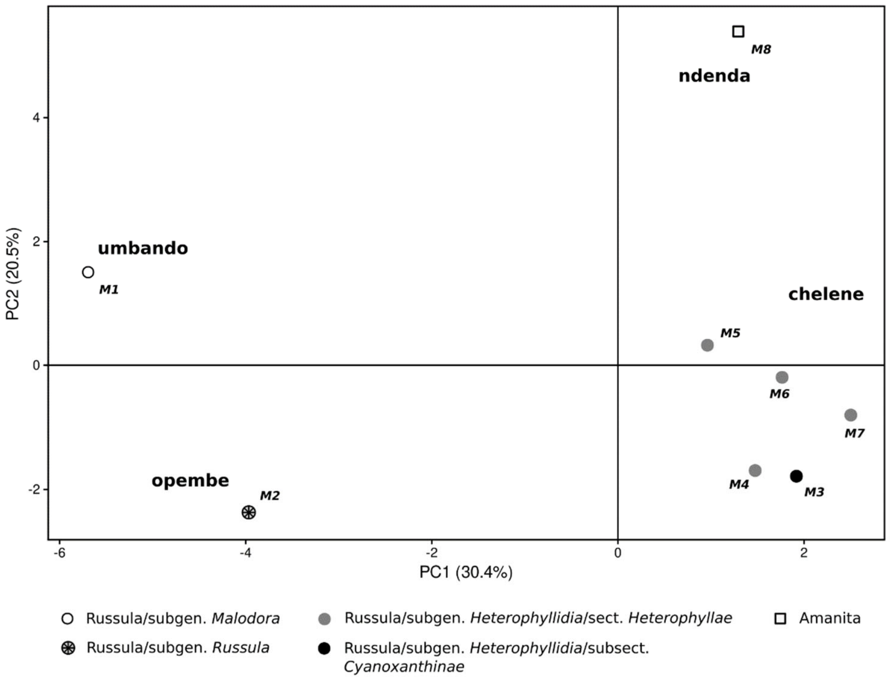

Figure S1. Principal Component Analysis (PCA) plot obtained with the nutritional and functional chemistry variables from Tables 3 to 7. Samples are marked with different symbols according to phylogenetic groups (Figure 3), and the local common names are included.
